# Molecular diagnostics of *Salmonella* and *Campylobacter* in human/animal fecal samples remain feasible after long-term sample storage without specific requirements

**DOI:** 10.3934/microbiol.2021024

**Published:** 2021-10-14

**Authors:** CB Harder, S Persson, J Christensen, A Ljubic, EM Nielsen, J Hoorfar

**Affiliations:** 1 Statens Serum institut, Dept. Bacteriology, Parasitology and Fungi, Artillerivej 5, 2300 Copenhagen, Denmark; 2 Molecular Ecology, Microbial Ecology and Evolutionary Genetics, Lund University, Sölvegatan 37, 223 62 Lund; 3 Danish Veterinary and Food Administration, Microbiological department, Søndervang 4, 4100 Ringsted; 4 AGC Biologics, Process Transfer, Vandtårnsvej 83, 2860 Søborg, Denmark; 5 Technical University of Denmark, National Food Institute, 2800 Kgs. Lyngby, Denmark

**Keywords:** Detection threshold, Campylobacter, Salmonella, gastritis, DNA stability, diagnostics, preservation methods, pathogens

## Abstract

Rapid advances in the development of sequencing technologies, numbers of commercial providers and diminishing costs have made DNA-based identification and diagnostics increasingly accessible to doctors and laboratories, eliminating the need for local investments in expensive technology and training or hiring of skilled technicians. However, reliable and comparable molecular analyses of bacteria in stool samples are dependent on storage and workflow conditions that do not introduce post-sampling bias, the most important factor being the need to keep the DNA at a stable detectable level. For that reason, there may remain other prohibitively costly requirements for cooling or freezing equipment or special chemical additives.

This study investigates the diagnostic detectability of *Salmonella* and *Campylobacter* DNA in human, pig and chicken stool samples, stored at different temperatures and with different preservation methods. Stool samples were spiked with 10^6^ CFU/mL of both *Salmonella* and *Campylobacter* strains stored at −20 °C, 5 °C and 20 °C (Room temperature, RT) and treated with either RNAlater, EDTA or Silica/ethanol. DNA was extracted at 9 different time points within 30 days and quantified by Qubit (total DNA) and qPCR (*Salmonella* and *Campylobacter* DNA). We found no statistically significant differences among the different preservation methods, and DNA from both species was easily detected at all time points and at all temperatures, both with and without preservation. This suggests that infections by these bacteria can be diagnosed and possibly also analysed in further detail simply by taking a stool sample in any suitable sealed container that can be transported to laboratory analysis without special storage or preservation requirements. We briefly discuss how this finding can benefit infection control in both developed and developing countries.

## 1. Introduction

Salmonellosis and Campylobacteriosis are two of WHO's four key causes of diarrhoea globally, and the leading causes of foodborne bacterial infections wordwide [1], with a combined estimated annual worldwide tally of cases of diarrhoea of between 100 and 200 million [2]. Nonetheless, in developing countries, infection with rota- or noroviruses surpass bacterial infections by a factor of more than two among the total food-related diarrhoeic diseases [3]. Indiscriminate use of antibiotics for ineffective treatment of diarrhoea without diagnosis in developing countries is a leading cause of the growing resistance to antibiotics [4]. A comparable overuse of antibiotics in livestock production in developed countries (often simply prophylactic, since *Salmonella* in pigs is most often asymptomatic [5], as is *Campylobacter* in chicken [6]) has further exacerbated the problem of antibiotic resistance [7]. The major problem is that antibiotics are cheap, while quality diagnostics is often inaccessible or prohibitively costly [4]. The dramatic advances in DNA sequencing and other molecular DNA-based techniques, increasing availability of commercial providers and decreasing costs has made molecular diagnostics more accessible and applicable for diagnostics of multiple types of infectious diseases, including in developing countries [8]–[11]. However, reliable sample quality often requires freezing, cold storage and transportation or chemical preservation, and this remains expensive and continues to be a major obstacle.

Both within veterinary and human clinical microbiology, stool specimens are essential to the diagnostics and typing of bacterial pathogens causing gastroenteritis. Conventional methods were based on culturing combined with serotyping and/or biochemical analyses [12]–[14], with PCR and conventional (Sanger) sequencing being increasingly applied [15]–[17]. Recently, culture-independent diagnostic tests (CIDTs), have gained ground at decentralized laboratories, as these are often faster, easier and less expensive. Fecal microbiome and metagenomic studies, in recent decades utilizing rapidly developing high throughput sequencing, have investigated the distribution and interplay of hundreds or thousands of bacteria simultaneously [18]–[22].

Many methodological parameters need to be considered when performing molecular analyses directly on stool samples, including sampling and storage, DNA extraction and molecular methods. Optimal sampling and storage ensures minimum changes in the quality and bacterial composition of species of interest, from the time of sampling until analysis. Conventional methods based on culturing were dependent on ambient and chemical conditions that would leave the bacteria cultivable while molecular methods are less demanding, as they only need relatively intact DNA to be successful. This area has recently gained renewed interest with microbiome and metagenomic studies, showing varying degrees of influence from this step. Consequently, several chemical preservation methods from human and veterinary field studies have been developed and tested for this specific purpose [23]–[27].

Procedures suited for molecular analyses for extracting DNA from stool samples, have also been intensively investigated. These include procedures for how to target DNA and remove critical components that have inhibitory effect on downstream analyses like PCR and sequencing. Many efficient in-house and commercial methods have been developed and compared in numerous studies [28]–[32]. Metagenomic studies have also shown that the observed bacterial species distribution varies with the chosen method of DNA extraction [33],[34].

A tremendous amount of molecular methods have been designed for a variety of purposes and new technologies are constantly being developed. Molecular methods are dependent on several critical reagents like primers, enzymes and co-factors and the method to choose depends on which answer is needed. Next generation sequencing is at present the most accelerating technology within this field and sequencing data per cost continues to increase. However, dealing with these technologies it is important to bear in mind that for example the chosen primers and sequence technology platforms may bias the data that could lead to misinterpretations [35].

In order to obtain molecular analyses directly on stool samples the above issues must be investigated and the impact from each individual step must be determined. This should allow the development of standardised method and comparison of data between different laboratories. Such initiatives would increase the quality and interpretation of metagenomic studies that are expected to increase in the future. The present study focuses on bacterial DNA preservation in stool samples from human, chicken and pigs, and investigates the diagnostic detectability of DNA of two important foodborne pathogens *Salmonella* and *Campyloba*cter in spiked samples treated with RNAlater, EDTA and ethanol/silica at room temperature (RT), 5 °C and −20 °C during a period of 30 days. The preservation methods were selected from the literature as being relatively inexpensive and easy to apply, containing few harmful chemicals and having a previously documented performance for stabilising nucleic acids [36]–[39].

## 2. Materials and methods

### 2.1. Experimental design

Human stool samples were spiked with both *Salmonella* and *Campylobacter*, chicken stool samples were spiked with *Campylobacter* and pig stool samples were spiked with *Salmonella*, all samples at 10^6^ CFU/mL. Additionally, one set of samples were prepared without spiking. Samples were treated with one of three different methods for preservation (EDTA, ethanol/silica and RNAlater) or left untreated (no preservation) and stored at three different temperatures; room temperature (20 °C, RT), 5 °C or −20 °C. DNA from each sample was extracted by Qiagen Fast Stool kit at the following time points (day): 0 (immediately after spiking), 1, 2, 3, 6, 10, 15, 20 and 30. Human samples were prepared in individual separate duplicates for each point in time and one DNA extraction was performed on each. Veterinary samples were prepared as single samples for each point in time and two DNA extractions were performed on the same sample at each . The extracted DNA was quantified by Qubit and analysed with species-specific qPCRs targeting *Campylobacter* and *Salmonella*. All tests were performed in duplicate, and presented as averages including standard deviations. Experiments on human and veterinary samples were performed at Statens Serum Institut (Copenhagen, Denmark) and the Technical University of Denmark (Søborg, Denmark), respectively.

### 2.2. Preparation of samples

Human stool samples were obtained from diarrhoeagenic patients who were culture and qPCR negative (see below) for both *Salmonella* and *Campylobacter*. Composite veterinary samples of fresh feces were randomly collected from a chicken flock and a pig herd with historical absence of *Campylobacter* and *Salmonella*, respectively, and tested negative by culturing. Prior to spiking, stool samples were mixed with physiological saline (0.9%) in the ratio of approximately 1:2 (w/vol) and homogenized with a Dispomix (Xiril). Human samples were spiked with in-house clinical strain *S. enterica* Brænderup (serotype H9812) and *C. jejuni* subsp. *jejuni* (CCUG 11284), chicken samples with in-house broiler strain *C. jejuni* DVI-SC181 and pig samples with *S. typhimurium* CCUG 31969. Prior to spiking, *Salmonella* strains were grown in nutrient broth and incubated at 37 °C and Campylobacter strains were grown in Mueller Hinton broth and incubated at 41.5 °C in a microaerobic atmosphere. Salmonella and Camplobacter were grown in liquid cultures to 107 cfu per mL as determined previously by triple plate counting, and from there diluted 10-fold in saline to obtain 106 to 102 cfu per mL for determination of standard curves. Ultimately, the liquid cultures were added to the homogenized stool samples at a final concentration of 106 CFU per mL stool sample. This spiking level was chosen for all samples, as for both genera it is known to be at the low end of the clinically relevant concentration spectrum [40],[41], a reliable detection limit for culture-based detection [42]; and allowing for a potentially substantial degradation while still remaining well above the 102/103 CFU per mL detection limit for our qPCR assays [43]–[45].

The spiked stool samples were aliquoted (250 µL) into 2 mL Eppendorf tubes and treated and mixed vigorously with either RNAlater® (Sigma-Aldrich) at the ratio 1:5 or 100 mM EDTA, pH 8 at the ratio 1:4 or 96% ethanol at the ratio 1:5, and finally one set of samples were left untreated. The ethanol treated samples were incubated for 24 hours at RT, the supernatant was carefully discarded by pouring, and silica gel beads (Sigma-Aldrich, type III, S7625) were added until the Eppendorf tubes were 3/4 full. Samples were stored at either RT, 5 °C or −20 °C until DNA extraction.

### 2.3. DNA extraction

Prior to DNA extraction the RNAlater treated samples were centrifuged at 5000 × *g* for 15 min, the supernatant was discarded and the pellet resuspended in 500 µL TE, sedimented at 500 × *g* for 15 min, and the pellet was used directly for DNA extraction. EDTA treated samples were centrifuged at 3000 × *g* for 15 min, and the pellet was used directly for DNA extraction. From the ethanol and silica treated samples, the beads were carefully poured off and the remaining material was used directly for DNA extraction. From samples without spiking and samples without preservation, 250 µL was used directly in the DNA extraction. All DNA extractions were performed by the QIAamp Fast Stool kit (Qiagen) by adding 1 mL InhibitEX and following the protocol for ‘pathogen detection’ when human samples were extracted and the protocol for ‘human DNA analysis' when veterinary samples were extracted. The main difference between the human DNA protocol and the pathogen detection protocol is the inclusion of a 5 minute 70 °C heating step to lyse more cells with thick walls (particularly gram-positive bacteria) in the latter. Products from both protocols eventually contain large amounts of human and bacterial DNA, and the differences concern only the relative bacterial DNA/total DNA ratios.

### 2.4. DNA analyses

The extracted DNA was quantified in duplicate on a Qubit® 2.0 Fluorometer (Life Technologies), using the Qubit® dsDNA High Sensitivity Assay Kit (Life Technologies). Real-time PCR for the detection of a 287 bp sequence of the 16S rRNA gene from *C. jejuni*, *C. coli* and *C. lari* was performed as described previously [45]. Real-time PCR for specific detection of *Salmonella* targeting a 94 bp region within the *ttr*RSBCA locus was performed as described previously [44]. PCR machines were Mx3005P (Startagene) and ABI7500 (Applied Biosystems) for veterinary and human samples, respectively. All runs included internal amplification controls and controls with no template, and all samples were analyzed in duplicate. Baseline values between runs were calibrated by including internal positive control templates.

### 2.5. Statistical analysis

We employ linear and logarithmic models to the absolute DNA content as well as to the Ct values measured over 0–30 days to test the decay of DNA over time, using R, version 3.4.3 [46]. Furthermore, we used the R package ‘Agricolae’ [47] to test the mean difference in the regression coefficients of the linear models for treatments, temperatures and sample type (human/chicken/pig *Salmonella*/*Campylobacter*). We used ANOVA for comparing the mean values, and applied Scheffe's post-hoc test to correct for multiple comparisons at a significance level of <0.05.

## 3. Results

A total of 540 DNA extractions were made from the following preparations: 5 different treatments, including: 3 preservation methods, one without preservation and one without preservation and spiking, 9 time points, 3 temperatures, two DNA extractions from each human stool sample (spiked simultaneously with both *Salmonella* and *Campylobacter*) and one from each of the chicken samples (spiked with *Campylobacter*) and pig samples (spiked with *Salmonella*). All samples were individual tubes kept separate, and after experiment initiation they were handled only once (i.e. when ultimately extracted), i.e there were no repeated measurements/extractions of the same vials successively.

DNA extracted from each of the 540 individual preparations was analysed by total DNA quantification and qPCR targeting species specific genes in *Salmonella* and *Campylobacter*, respectively. Total DNA quantification and qPCR data are shown on Figure 1 A + B + Figure 2 A + B (no preservation and EDTA) and Figure 1 C + D + Figure 2 C + D (ethanol/silica and RNAlater). All non-spiked samples were qPCR negative, i.e. no amplification at all (data not shown).

**Figure 1 A-B.**
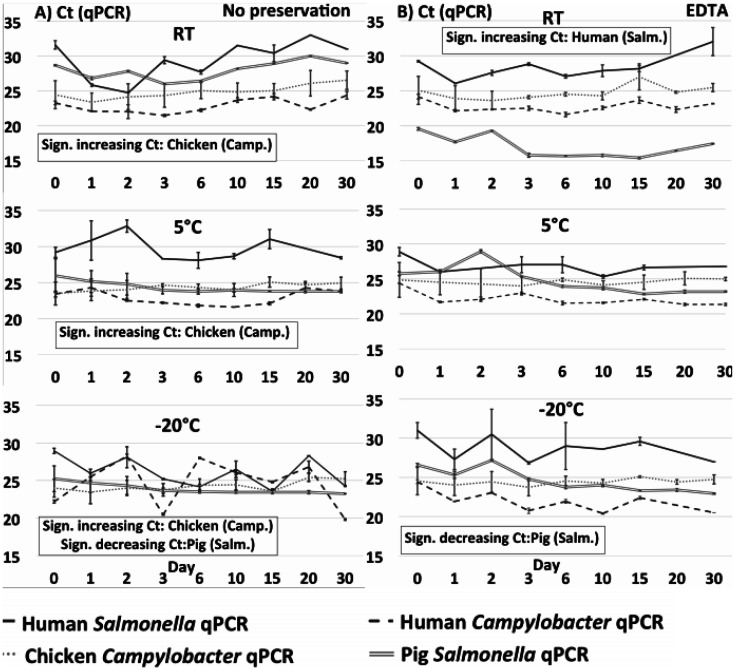
qPCR on spiked stool samples prepared without preservation (A) and preserved with EDTA (B) at 9 time points during 30 days stored at either RT, 5 °C or −20 °C. Significance threshold at 0.05. Note that an increasing Ct means declining DNA content, and vice versa.

**Figure 1 C-D.**
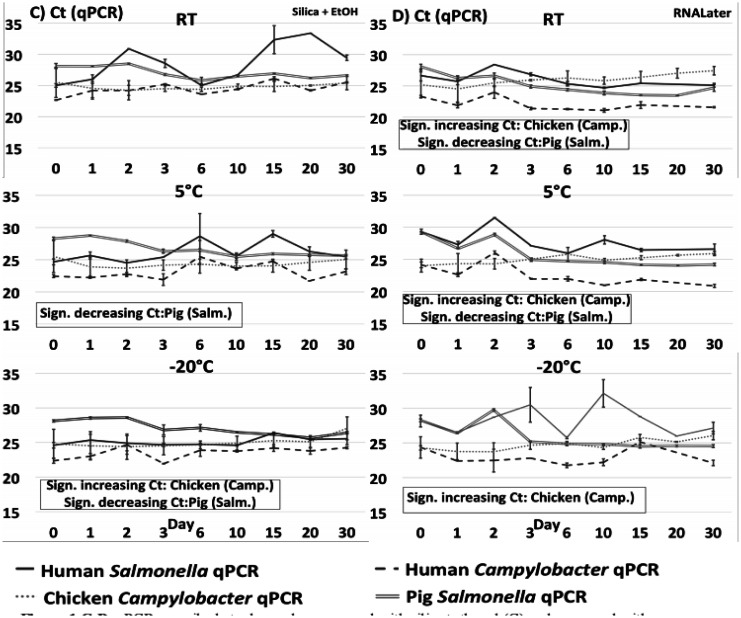
qPCR on spiked stool samples preserved with silica+ethanol (C) and preserved with RNALater (D) at 9 time points during 30 days stored at either RT, 5 °C or −20 °C. Significance threshold at 0.05. Note that an increasing Ct means declining DNA content, and vice versa.

**Figure 2 A-B.**
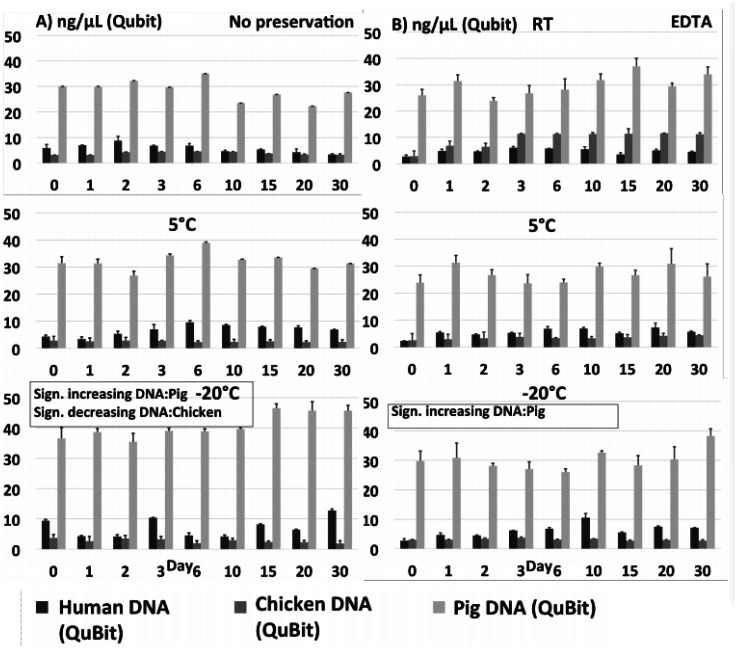
Total DNA quantification on spiked stool samples prepared without preservation (Panel A) and preserved with EDTA (Panel B) at 9 time points during 30 days stored at either RT, 5 °C or −20 °C. Significance threshold at 0.05.

**Figure 2 C-D.**
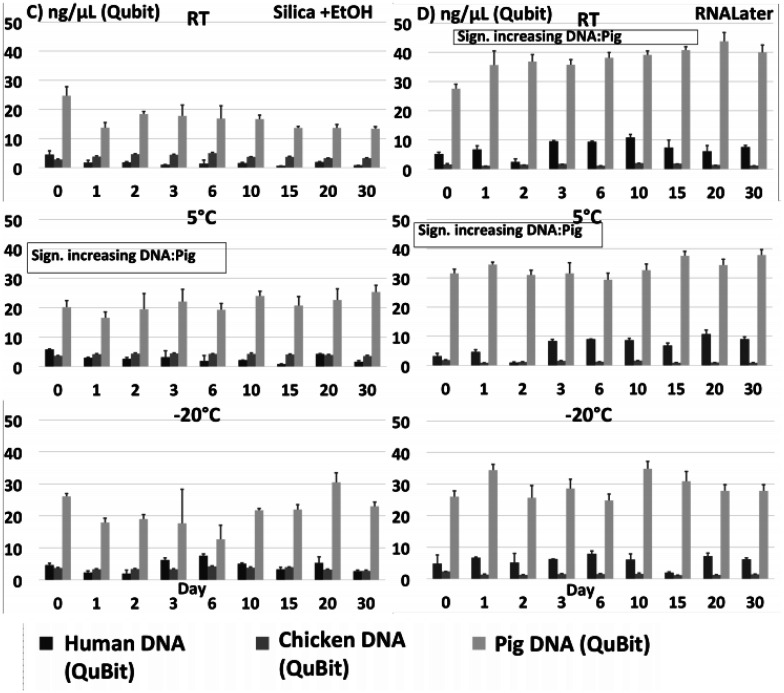
Total DNA quantification on spiked stool samples preserved with silica+ethanol (C) and preserved with RNALater (D) at 9 time points during 30 days stored at either RT, 5 °C or −20 °C. Significance threshold at 0.05.

Quantification of total DNA displayed a few fluctuations within a few time series, and these sometimes coincided with corresponding variation in qPCR, indicating that some extractions may have been relatively less efficient, or the homogenization prior to spiking incomplete. Comparing the different matrices, we observed a relatively high amount of DNA extracted from pig stool (average 28,9 ng/µL) followed by human (average 5,4 ng/µL) and chicken (average 3,8 ng/µL) stool. Low yield from chickens could be explained by a significant content of complex and non-degraded plant material, while high yield from pig samples may be related to the use of the ‘human DNA analysis' protocol, which is designed to extract more DNA. Most Ct curves except those treated with ethanol and silica had a small decline in Ct during the first day, most likely due to continued growth of the two spiking cultures. Regarding samples at 5 °C and −20 °C, this growth may have taken place before the sample reached the final storage temperature and the eventual cooling/freezing effect set in.

In order to interpret the qPCR data during the time course, we used the gradient obtained by linear regression of the 9 time points from each of the 48 data sets (3 preservation methods/one without preservation, 3 temperatures and 4 host/bacteria combinations). Note that increasing Ct values correspond to a decrease in target DNA during the time course and vice versa for low gradient values. See Table S1 for adjusted R^2^- and p- values. We report the Ct values instead of concentrations (cfu/mL), since we did not have standard curves for all samples which would allow a conversion of Ct values to concentrations (cfu/mL); however, since we compare the rates of change/coefficients of increase/decrease over time, this makes no difference to the results, as the relative differences are identical. See Figures. S1 + S2 for the Human *Campylobacter* data presented as cfu/mL values calculated from a standard curve.

**Figure 3.**
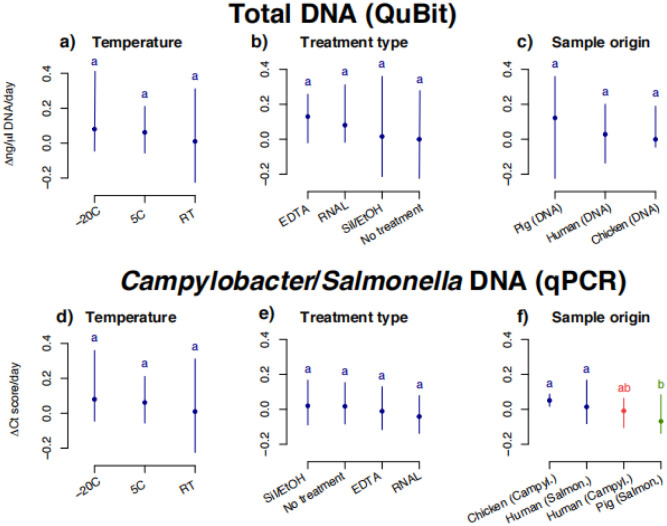
Average slopes/regression coefficients for total DNA (a, b, c), and Ct qPCR values (d, e, f) over the 30 days. Difference between means analysed using ANOVA and Scheffe's honestly significant difference test for multiple comparisons at significance level at a < 0.05. In all graphs, significantly different groups and ranges are indicated by the letters a–b, meaning that groups sharing the same letter are not significantly different from each other.

There were some slight but significant differences between individual series/samples: *Salmonella* in pig samples had a small but statistically significant increase in DNA (i.e., a decrease in Ct values, Figures 1 + 2) measured by qPCR for all samples (except “no preservation” and EDTA at 5 °C), indicating either a small proliferation of *Salmonella* bacteria, or non-target DNA and/or PCR inhibitors being degraded during the time course resulting in qPCR with lower Ct's. There was a slight, significantly higher rate of decrease in detectable *Salmonella* target DNA in the pig samples as compared to the human and chicken samples (Figure 3f), which we did not observe in the total DNA content (Figure 3c).

The opposite was observed in *Campylobacter* in chicken samples, i.e. in all samples except those treated with ethanol and silica at 5°C and RT we observed a small but statistically significant decline in *Campylobacter* DNA during the time course. In human samples, the only statistical differences we observed for the duration of the experiment were a slight decline in total DNA for the ‘no preservation’ at RT, and a slight increase in *Salmonella* DNA for the EDTA sample at RT.

Overall, the most important result was the relatively small changes over the 30-days for all four sample types, regardless of temperature or (lack of) preservation method. Most of the reported differences over time found in Table S1 and described above were small and unsystematic, and in the comparisons of all slopes in the linear models divided into preservation methods, temperatures and sample type (human/chicken/pig *Salmonella*/*Campylobacter*) (Figure 3), we did not find any statistically significant differences in the changes in DNA concentrations/Ct values between any of the storage temperatures (Figure 3a, 3d) nor between the different preservation methods (Figure 3b, 3e). While there was a significantly stronger degradation of particularly *Campylobacter* DNA in the chicken samples compared to the other series (Figure 3f), the pathogen DNA still remained clearly detectable in chicken stools by qPCR throughout all 30 days.

## 4. Discussion

CIDTs, metagenomic and microbiome studies targeting DNA purified directly from clinical samples need DNA of a sufficient quality for the desired purpose. DNA quality depends on sample collection procedure, storage condition and DNA extraction method, and ideally, each of these steps should be optimized individually for best final outcome. However, costly optimization is not realistic in many circumstances. For example, freezing is generally considered an optimal way of preserving DNA [37], but for practical reasons, veterinary samples collected directly by farmers themselves as well as human samples from non-hospitalized patients are often transported or shipped to centralized laboratories in a native state and exposed to RT that may take several days. For developing countries, lack of access to quality cold storage and cold chain transportation is a major barrier to all types of centralised sample analysis, vaccine distributions or food safety [48]–[50]. While chemical preservation of DNA before analysis can be an easier/cheaper strategy for increasing the quality of downstream analyses than cold storage, price and availability of preservation supplies can still remain a major challenge in many places in the developing world. This project was initiated to investigate the effects of both temperature and common preservation methods for preservation of DNA from sampling to diagnostics of two widespread gut pathogens. Even over a fairly long time window of 30 days, we did not observe any major benefits from the chemicals nor the storage temperatures for our bacterial detection. In fact, both *Campylobacter* and *Salmonella* DNA was detected in all matrices of non-preserved samples at all time points and temperatures at approximately similar levels, indicating limited degradation of these species in native feces.

Empirically, we had previous experience that the human DNA extraction protocol had a higher overall DNA yield efficiency for veterinary samples, and the comparatively higher bacterial yield of the bacterial/pathogen protocol with heat lysis was more important for retrieving gram-positive bacterial DNA (both our pathogens are gram-negative). While there is no indication that the different extraction protocols made any difference to detectability over time, the recommendation must be to use the pathogen protocol also for veterinary samples in the future.

On the very practical level, we found the ethanol/silica protocol (first soaking in ethanol, and then adding silica-beads) to be time-consuming and somewhat impractical, as the dried sample material stuck to the beads and made them difficult to remove before DNA extraction without sample loss, and removing the preservation agent (i.e. the supernatant) without further loss of sample also required great effort. Removing the supernatant also required considerable care for samples treated with RNAlater, as the fecal sample was hard to sediment even after two centrifugations. In comparison, EDTA preservation method did not present any technical difficulties. In terms of temperature, freezing untreated stool samples may lyse cells and lead to DNA degradation during the extraction process, and thus decrease yields compared to above-zero temperature storages [34],[51]. The frequently encountered problem with maintaining intact frozen-storage transport chains and freeze-thaw interchanges in the process from sampling to extraction will exacerbate this problem. Thus, even if frozen storage may still be accessible to clinicians that otherwise have limited budgets that exclude purchases of expensive chemical preservatives, the general recommendation must still be against freezing untreated stool samples.

We wish to stress that our focus rested on the specific issue of diagnostics of our two pathogens in fecal matter over a realistic timeframe in clinical work from sampling to medical/microbiological analysis. While we must consider silica desiccation as generally unsuitable for preservation of fecal/viscous samples, EDTA would have advantages in preserving longer (e.g + 1000 bp) fragments and longer-term storage [52], and RNAlater could be valuable for the emerging field of fecal transcriptomics analyses [53]. Furthermore, for other increasingly ubiquitous DNA-based studies on fecal matter (and human/bodily samples in general) as general community composition analyses, immediate fixation upon sampling and subsequent cold storage of samples remain essential, even as the commonly applied fixation method of freezing raw samples [54]–[56] remains ill-advised if not done in combination with another sample treatment [51].

But while we certainly do not suggest a correction to the CDC general recommendations for stool sample handling [57], our results do suggest that simply sampling fecal matter (with normally reasonable considerations to avoid cross-contamination) in a clean sealable container, and storing/shipping under room temperature conditions, is indeed sufficient for DNA-based diagnostic purposes involving *Campylobacter* and *Salmonella* infections, the most widespread gastroenteric bacterial pathogens, for typical samples. Furthermore, as we targeted fragments of up to 300 bp in the *Campylobacter* assay, the stable existence of fragments of this size over 30 days also means that the required lengths for multilocus sequence typing [17] or shotgun metagenomic detection [58] of these pathogens are also within reach. Application of molecular/DNA-based diagnostics is increasing in the developing world [59], and during the recent COVID19 pandemic, we have witnessed an implementation of PCR-based testing all across the world (e.g, with tens of millions of RT-PCR tests taken in less than a year in India [60]) that is probably unsurpassed in scale and size. We suggest that many more fecal samples could and should be analysed for diagnosis of *Salmonella* or *Campylobacter* and probably also other bacterial causes of gastroenteritis that presently simply do not get taken because normal optimal sample handling is a priori believed to be impractical. This would also apply to veterinary sampling of stools from seemingly unaffected livestock.

Lack of proper diagnosis separating bacterial from viral causes of diarrhoea across the developing world remain a major issue [61]. Remediation of this could lead to a substantial reduction in the use of antibiotics[4],[7], which is a globally shared concern for developing and developed countries alike. Knowing that reliable DNA-based diagnostic analysis can be done on fecal samples which can be taken at low cost at any point-of-use and transported to central facilities for analysis is one step in that direction.

## References

[1] WHO (2015). WHO estimates of the global burden of foodborne diseases: foodborne disease burden epidemiology reference group 2007–2015.

[2] Chlebicz A, Śliżewska K (2018). Campylobacteriosis, Salmonellosis, Yersiniosis, and Listeriosis as zoonotic foodborne diseases: A Review. Int J EnvironRes Public Health.

[3] Fletcher SM, McLaws ML, Ellis JT (2013). Prevalence of gastrointestinal pathogens in developed and developing countries: systematic review and meta-analysis. J Public Health Res.

[4] Chokshi A, Sifri Z, Cennimo D (2019). Global contributors to antibiotic resistance. J Global Infect Dis.

[5] Bonardi S (2017). *Salmonella* in the pork production chain and its impact on human health in the European Union. Epidemiol Infect.

[6] Awad WA, Molnár A, Aschenbach JR (2014). *Campylobacter* infection in chickens modulates the intestinal epithelial barrier function. Innate Immunity.

[7] Landers TF, Cohen B, Wittum TE (2012). A review of antibiotic use in food animals: perspective, policy, and potential. Public Health Rep.

[8] Weerakoon K, Gordon C, McManus D (2018). DNA diagnostics for schistosomiasis control. Tropical Med Infect Dis.

[9] Schultze A, Akmatov MK, Andrzejak M (2014). Comparison of stool collection on site versus at home in a population-based study. Bundesgesundheitsblatt Gesundheitsforschung Gesundheitsschutz.

[10] Platts-Mills JA, Liu J, Gratz J (2014). Detection of *Campylobacter* in stool and determination of significance by culture, enzyme immunoassay, and PCR in developing countries. J Clin Microbiol.

[11] Büscher P, Deborggraeve S (2015). How can molecular diagnostics contribute to the elimination of human African trypanosomiasis?. Expert Rev Mol Diagn.

[12] Love BC, Rostagno MH (2008). Comparison of five culture methods for *Salmonella* isolation from swine fecal samples of known infection status. J Vet Diagn Invest.

[13] Buss JE, Thacker E, Santiago M (2020). Culture methods to determine the limit of detection and survival in transport media of *Campylobacter Jejuni* in human fecal specimens. J Vis Exp.

[14] Herikstad H, Motarjemi Y, Tauxe RV (2002). Salmonella surveillance: a global survey of public health serotyping. Epidemiol Infect.

[15] Bale J, Meunier D, Weill FX (2016). Characterization of new *Salmonella serovars* by whole-genome sequencing and traditional typing techniques. J Med Microbiol.

[16] Bereswill S, Jerome JP, Bell JA (2011). Standing genetic variation in contingency loci drives the rapid adaptation of *Campylobacter jejuni* to a novel host. PLoS One.

[17] Achtman M, Wain J, Weill FX (2012). Multilocus sequence typing as a replacement for serotyping in *Salmonella enterica*. PLoS Pathogens.

[18] Turnbaugh PJ, Ley RE, Hamady M (2007). The human microbiome project. Nature.

[19] Qin J, Li R, Raes J (2010). A human gut microbial gene catalogue established by metagenomic sequencing. Nature.

[20] Leser TD, Amenuvor JZ, Jensen TK (2002). Culture-independent analysis of gut bacteria: the pig gastrointestinal tract microbiota revisited. Appl Environ Microbiol.

[21] Gill SR, Pop M, DeBoy RT (2006). Metagenomic analysis of the human distal gut microbiome. Science.

[22] Faith JJ, Guruge JL, Charbonneau M (2013). The long-term stability of the human gut microbiota. Science.

[23] Jenkins SV, Vang KB, Gies A (2018). Sample storage conditions induce post-collection biases in microbiome profiles. BMC Microbiol.

[24] Choo JM, Leong LEX, Rogers GB (2015). Sample storage conditions significantly influence faecal microbiome profiles. Sci Rep.

[25] Anderson EL, Li W, Klitgord N (2016). A robust ambient temperature collection and stabilization strategy: Enabling worldwide functional studies of the human microbiome. Sci Rep.

[26] Wu GD, Lewis JD, Hoffmann C (2010). Sampling and pyrosequencing methods for characterizing bacterial communities in the human gut using 16S sequence tags. BMC Microbiol.

[27] Hale VL, Tan CL, Knight R (2015). Effect of preservation method on spider monkey (Ateles geoffroyi) fecal microbiota over 8weeks. J Microbiol Methods.

[28] Claassen S, du Toit E, Kaba M (2013). A comparison of the efficiency of five different commercial DNA extraction kits for extraction of DNA from faecal samples. J Microbiol Methods.

[29] Kongmuang U, Luk JM, Lindberg AA (1994). Comparison of three stool-processing methods for detection of *Salmonella* serogroups B, C2, and D by PCR. J Clinical Microbiol.

[30] McOrist AL, Jackson M, Bird R (2002). A comparison of five methods for extraction of bacterial DNA from human faecal samples. J Microbiol Methods.

[31] Persson S, de Boer RF, Kooistra-Smid AMD (2011). Five commercial DNA extraction systems tested and compared on a stool sample collection. Diagn Microbiol Infect Dis.

[32] Rapp D (2010). DNA extraction from bovine faeces: current status and future trends. J Appl Microbiol.

[33] Wesolowska-Andersen A, Bahl MI, Carvalho V (2014). Choice of bacterial DNA extraction method from fecal material influences community structure as evaluated by metagenomic analysis. Microbiome.

[34] Bahl MI, Bergström A, Licht TR (2012). Freezing fecal samples prior to DNA extraction affects the Firmicutes to Bacteroidetes ratio determined by downstream quantitative PCR analysis. FEMS Microbiol Lett.

[35] White BA, Clooney AG, Fouhy F (2016). Comparing apples and oranges?: next generation sequencing and its impact on microbiome analysis. Plos One.

[36] Nsubuga AM, Robbins MM, Roeder AD (2004). Factors affecting the amount of genomic DNA extracted from ape faeces and the identification of an improved sample storage method. Mol Ecol.

[37] Vlčková K, Mrázek J, Kopečný J (2012). Evaluation of different storage methods to characterize the fecal bacterial communities of captive western lowland gorillas (Gorilla gorilla gorilla). J Microbiol Methods.

[38] Wasser SK, Houston CS, Koehler GM (1997). Techniques for application of faecal DNA methods to field studies of Ursids. Mol Ecol.

[39] Nechvatal JM, Ram JL, Basson MD (2008). Fecal collection, ambient preservation, and DNA extraction for PCR amplification of bacterial and human markers from human feces. J Microbiol Methods.

[40] Bennett JE, Dolin R, Blaser MJ (2014). Mandell, douglas, and bennett's principles and practice of infectious diseases: 2-volume set (Vol. 2). Elsevier Health Sciences.

[41] Anderson NW, Buchan BW, Ledeboer NA (2014). Comparison of the BD MAX enteric bacterial panel to routine culture methods for detection of *Campylobacter, Enterohemorrhagic Escherichia coli (O157), Salmonella*, and *Shigella* isolates in preserved stool specimens. J Clini Microbiol.

[42] Buss JE, Cresse M, Doyle S (2019). *Campylobacter* culture fails to correctly detect *Campylobacter* in 30% of positive patient stool specimens compared to non-cultural methods. European J Clini Microbiol Infect Dis.

[43] Lund M, Nordentoft S, Pedersen K (2004). Detection of *Campylobacter* spp. in chicken fecal samples by real-time PCR. J Clin Microbiol.

[44] Josefsen MH, Krause M, Hansen F (2007). Optimization of a 12-hour TaqMan PCR-based method for detection of *Salmonella* bacteria in meat. Appl Environ Microbiol.

[45] Josefsen MH, Löfström C, Hansen TB (2010). Rapid quantification of viable *Campylobacter* bacteria on chicken carcasses, using real-time PCR and propidium monoazide treatment, as a tool for quantitative risk assessment. Appl Environ Microbiol.

[46] (2017). R Core Team: A language and environment for statistical computing. R Foundation for Statistical Computing, Vienna, Austria.

[47] de Mendiburu F (2019). Package ‘agricolae’. R Package, Version, 1–2.

[48] Songane M (2017). Challenges for nationwide vaccine delivery in African countries. Int J Health Econ Manage.

[49] Yahia EM (2009). Cold chain development and challenges in the developing world. VI International Postharvest Symposium.

[50] Oura CAL, Edwards L, Batten CA (2013). Virological diagnosis of African swine fever—comparative study of available tests. Virus Res.

[51] Dorsaz S, Charretier Y, Girard M (2020). Changes in microbiota profiles after prolonged frozen storage of stool suspensions. Front Cell Infect Microbiol.

[52] Milde A, Haas-Rochholz H, Kaatsch HJ (1999). Improved DNA typing of human urine by adding EDTA. Int J Legal Med.

[53] Fremin BJ, Bhatt AS (2020). A combined RNA-Seq and comparative genomics approach identifies 1,085 candidate structured RNAs expressed in human microbiomes. BioArxiv.

[54] The Human Microbiome Project Consortium (2012). A framework for human microbiome research. Nature.

[55] Frølund M, Wikström A, Lidbrink P (2018). The bacterial microbiota in first-void urine from men with and without idiopathic urethritis. Plos One.

[56] Jung CE, Chopyk J, Shin JH (2019). Benchmarking urine storage and collection conditions for evaluating the female urinary microbiome. Sci Rep.

[57] Prevention CfDCa (2021). Guidelines for Specimen Collection: Instructions for Collecting Stool Specimens.

[58] Andersen SC, Kiil K, Harder CB (2017). Towards diagnostic metagenomics of Campylobacter in fecal samples. BMC Microbiol.

[59] Abou Tayoun AN, Burchard PR, Malik I (2014). Democratizing Molecular Diagnostics for the Developing World. Am J Clin Pathol.

[60] Indian Council of Medical Research.

[61] Parashar UD, Nelson EAS, Kang G (2013). Diagnosis, management, and prevention of rotavirus gastroenteritis in children. BMJ.

