# Supplementary material for: Molecular diagnostics of Salmonella and Campylobacter in human/animal fecal samples remain feasible after long-term sample storage without specific requirements
Source: AIMS Microbiol. 2021 Oct 14;7(4):399–414. doi: 10.3934/microbiol.2021024 (PMC8712530; doi:10.3934/microbiol.2021024)
Supplement: Supplementary file 1 [file microbiol-07-04-024-s001.pdf]

Microbiology

AIMS Microbiology, 7(4): 399–414.
DOI: 10.3934/microbiol.2021024
Received: 17 March 2021
Accepted: 05 September 2021
Published: 14 October 2021

http://www.aimspress.com/journal/microbiology

Research article

Molecular diagnostics of *Salmonella* and *Campylobacter* in human/animal fecal samples remain feasible after long-term sample storage without specific requirements

Harder CB[1,2,*], Persson S[1], Christensen J[3], Ljubic A[4], Nielsen EM[1] and Hoorfar J[5]

1. Statens Serum institut, Dept. Bacteriology, Parasitology and Fungi, Artillerivej 5, 2300 Copenhagen, Denmark
2. Molecular Ecology, Microbial Ecology and Evolutionary Genetics, Lund University, Sölvegatan 37, 223 62 Lund
3. Danish Veterinary and Food Administration, Microbiological department, Søndervang 4, 4100 Ringsted
4. AGC Biologics, Process Transfer, Vandtårnsvej 83, 2860 Søborg, Denmark
5. Technical University of Denmark, National Food Institute, 2800 Kgs. Lyngby, Denmark.

* **Correspondence:** Email: cbharder@bio.ku.dk.

Supplementary

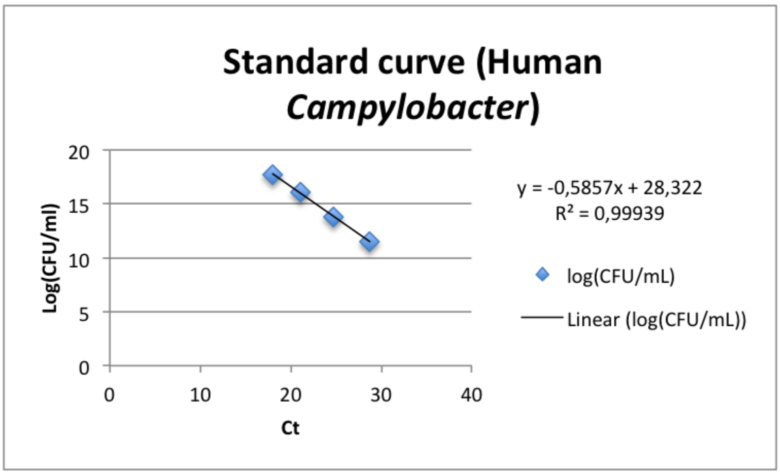

Figure S1. The standard curve for the Human *Campylobacter* qPCR assay used for calculations of cfu values in Figure S2.

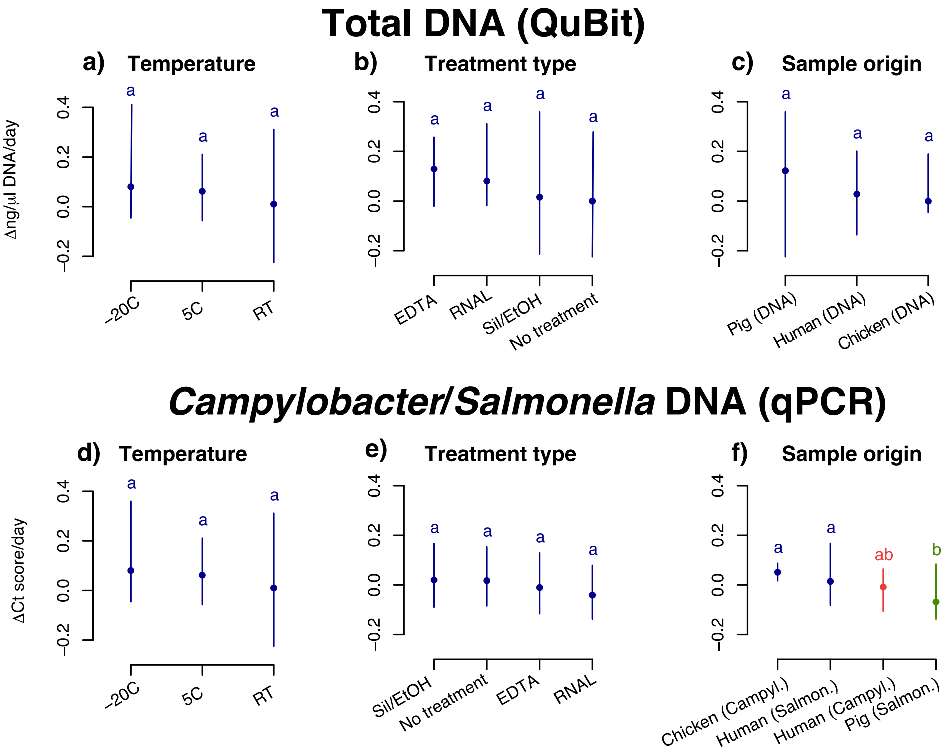

Figure S2. Average slopes/regression coefficients for total DNA (a,b,c), and Ct qPCR values (d,e,f) over the 30 days. Difference between means analysed using ANOVA and Scheffe´s honestly significant difference test for multiple comparisons at significance level at < 0.05. In all graphs, significantly different groups and ranges are indicated by the letters a–b, meaning that groups sharing the same letter are not significantly different from each other.

Table S1. Linear models of total DNA (DNA) and Ct values (Salm./Camp.) as a function of time over the 30-day timeframe. Significant decreases/increases at =0.05 **in bold**. Note the near-complete lack of obvious changes in all human samples.

Treatment	Sample type	Temperature	R^2	p-value
Silica/EtOH	Human(DNA)	RT	0.1097	0.2017
Silica/EtOH	Human(Salm.)	RT	0.1929	0.1317
Silica/EtOH	Human(Camp.)	RT	0.2139	0.1179
Silica/EtOH	Chicken(DNA)	RT	0.03175	0.2983
Silica/EtOH	Chicken(Camp.)	RT	0.1361	0.1764
Silica/EtOH	Pig(DNA)	RT	0.3121	0.06845
Silica/EtOH	Pig(Salm.)	RT	0.2066	0.1225
Silica/EtOH	Human(DNA)	5 ℃	0.03189	0.298
Silica/EtOH	Human(Salm.)	5 ℃	-0.08756	0.5696
Silica/EtOH	Human(Camp.)	5 ℃	-0.1288	0.7763
Silica/EtOH	Chicken(DNA)	5 ℃	0.2252	0.111
Silica/EtOH	Chicken(Camp.)	5 ℃	-0.04783	0.4518
**Silica/EtOH**	**Pig(DNA)**	**5 ℃**	**0.4844**	**0.0224**
**Silica/EtOH**	**Pig(Salm.)**	**5 ℃**	**0.4691**	**0.025**
Silica/EtOH	Human(DNA)	−20 ℃	-0.1242	0.7433
Silica/EtOH	Human(Salm.)	−20 ℃	0.2244	0.1115
Silica/EtOH	Human(Camp.)	−20 ℃	0.1496	0.1647
Silica/EtOH	Chicken(DNA)	−20 ℃	0.06559	0.2516
**Silica/EtOH**	**Chicken(Camp.)**	**−20 ℃**	**0.7686**	**0.00119**
Silica/EtOH	Pig(DNA)	−20 ℃	0.0849	0.2284
**Silica/EtOH**	**Pig(Salm.)**	**−20 ℃**	**0.5021**	**0.0196**
RNALater	Human(DNA)	RT	-0.1123	0.6743
RNALater	Human(Salm.)	RT	0.1863	0.1578
RNALater	Human(Camp.)	RT	0.01014	0.3405
RNALater	Chicken(DNA)	RT	-0.1226	0.7329
**RNALater**	**Chicken(Camp.)**	**RT**	**0.7733**	**0.0011**
**RNALater**	**Pig(DNA)**	**RT**	**0.4192**	**0.03529**
RNALater	Pig(Salm.)	RT	0.304	0.0717
RNALater	Human(DNA)	5 ℃	0.319	0.06575
RNALater	Human(Salm.)	5 ℃	0.0745	0.2577
RNALater	Human(Camp.)	5 ℃	0.2664	0.1089
RNALater	Chicken(DNA)	5 ℃	0.1562	0.1592
**RNALater**	**Chicken(Camp.)**	**5 ℃**	**0.5536**	**0.01304**
**RNALater**	**Pig(DNA)**	**5 ℃**	**0.4693**	**0.02499**
**RNALater**	**Pig(Salm.)**	**5 ℃**	**0.39**	**0.04266**
RNALater	Human(DNA)	−20 ℃	-0.1423	0.9531
RNALater	Human(Salm.)	−20 ℃	-0.09789	0.6089
RNALater	Human(Camp.)	−20 ℃	-0.1611	0.8711
RNALater	Chicken(DNA)	−20 ℃	0.05355	0.2673
**RNALater**	**Chicken(Camp.)**	**−20 ℃**	**0.6849**	**0.00362**

Continued on next page

Treatment	Sample type	Temperature	R^2	p-value
RNALater	Pig(DNA)	−20 ℃	-0.1428	0.9833
RNALater	Pig(Salm.)	−20 ℃	0.3075	0.07029
**No treatment**	**Human(DNA)**	**RT**	**0.6444**	**0.00563**
No treatment	Human(Salm.)	RT	0.2095	0.1207
No treatment	Human(Camp.)	RT	0.3008	0.07306
No treatment	Chicken(DNA)	RT	-0.04699	0.4497
**No treatment**	**Chicken(Camp.)**	**RT**	**0.8224**	**0.00046**
No treatment	Pig(DNA)	RT	0.1954	0.1299
No treatment	Pig(Salm.)	RT	0.3251	0.06344
No treatment	Human(DNA)	5 ℃	0.07125	0.2446
No treatment	Human(Salm.)	5 ℃	-0.08067	0.5154
No treatment	Human(Camp.)	5 ℃	-0.06705	0.5035
No treatment	Chicken(DNA)	5 ℃	0.3218	0.06469
**No treatment**	**Chicken(Camp.)**	**5 ℃**	**0.467**	**0.0254**
No treatment	Pig(DNA)	5 ℃	-0.135	0.8316
No treatment	Pig(Salm.)	5 ℃	0.3314	0.06112
No treatment	Human(DNA)	−20 ℃	0.1185	0.1929
No treatment	Human(Salm.)	−20 ℃	-0.003396	0.3568
No treatment	Human(Camp.)	−20 ℃	-0.0558	0.4722
**No treatment**	**Chicken(DNA)**	**−20 ℃**	**0.4165**	**0.03591**
**No treatment**	**Chicken(Camp.)**	**−20 ℃**	**0.5147**	**0.01782**
**No treatment**	**Pig(DNA)**	**−20 ℃**	**0.7346**	**0.001943**
**No treatment**	**Pig(Salm.)**	**−20 ℃**	**0.455**	**0.02765**
EDTA	Human(DNA)	RT	-0.1427	0.9773
**EDTA**	**Human(Salm.)**	**RT**	**0.4632**	**0.03785**
EDTA	Human(Camp.)	RT	-0.1217	0.7273
EDTA	Chicken(DNA)	RT	0.2743	0.08488
EDTA	Chicken(Camp.)	RT	0.1916	0.1326
EDTA	Pig(DNA)	RT	0.3171	0.06647
EDTA	Pig(Salm.)	RT	-0.02663	0.4029
EDTA	Human(DNA)	−20 ℃	0.1247	0.187
EDTA	Human(Salm.)	−20 ℃	-0.00272	0.3602
EDTA	Human(Camp.)	−20 ℃	0.1631	0.1751
EDTA	Chicken(DNA)	−20 ℃	0.2955	0.07532
EDTA	Chicken(Camp.)	−20 ℃	0.1569	0.1587
**EDTA**	**Pig(DNA)**	**−20 ℃**	**0.4066**	**0.03833**
**EDTA**	**Pig(Salm.)**	**−20 ℃**	**0.5671**	**0.01163**

AIMS Press

© 2021 the Author(s), licensee AIMS Press. This is an open access article distributed under the terms of the Creative Commons Attribution License (http://creativecommons.org/licenses/by/4.0)
